# Successful reverse cannulation and needle-knife papillotomy of the minor papilla for accessory pancreatic duct cannulation

**DOI:** 10.1055/a-2573-7409

**Published:** 2025-04-29

**Authors:** Yan Zhang, Yuping Zhang, Shanbin Wu, Qing Yan, Jielei Li, Guoliang Zhao

**Affiliations:** 1 66310 Department of Gastroenterology, The First Affiliated Hospital of Shandong First Medical University & Shandong Provincial Qianfoshan Hospital, Jinan, China

Pancreas divisum is the most common congenital malformation of the pancreas. It usually causes no symptoms or complications, but a small percentage of persons with this malformation develop recurrent acute pancreatitis. Patients with recurrent acute pancreatitis may benefit from endoscopic sphincterotomy of the minor papilla to open up the outflow of the dorsal pancreatic duct 1 2 . Here, we describe a case of pancreas divisum that was treated with reverse cannulation and needle-knife papillotomy of the minor papilla and placement of a pancreatic duct stent.

A 50-year-old man presented with recurrent acute pancreatitis. Pancreas divisum was diagnosed using endoscopic ultrasound and magnetic resonance cholangiopancreatography. The pancreatogram revealed that the main pancreatic duct (MPD) was bifurcated and the branched pancreatic duct was slender ( Fig. 1 ).

**Fig. 1:**
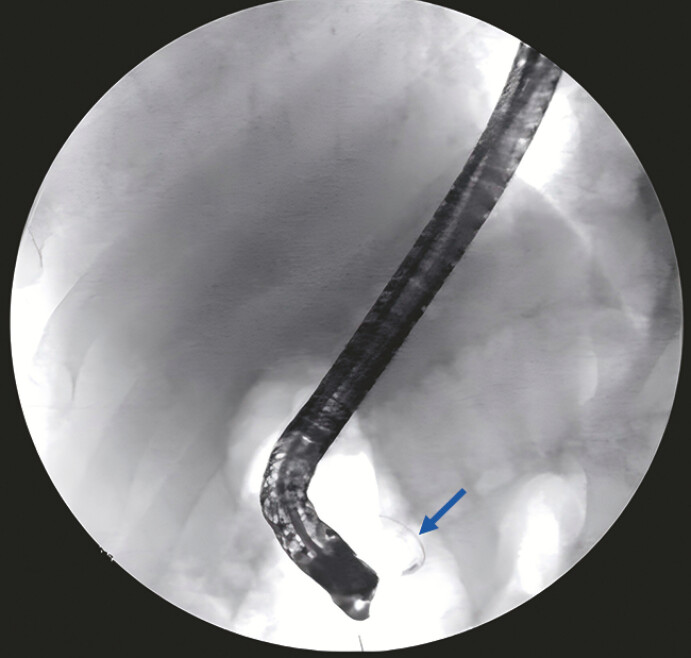
Pancreatogram revealed that the MPD was bifurcated and the branched pancreatic duct was slender (blue arrow). MPD, main pancreatic duct.

The minor papilla was not obvious under the microscope ( Fig. 2 ). The operator made several attempts to cannulate the minor papilla, all of which were unsuccessful due to the inconspicuous minor papilla orifice. Eventually, the guidewire (450 cm, Jagwire; Boston Scientific Corp., Marlborough, Massachusetts, USA) in the MPD successfully passed through the minor papilla and coiled in the duodenal lumen ( Fig. 3 **a, b** ). Then, under the guidance of the guide wire, the approximate location of the minor papilla was found, and minor papilla sphincterotomy was performed using the needle knife after pulling out the guide wire. Then, the guidewire intubated the minor papilla along the incision in the accessory pancreatic duct (APD; 450 cm, Jagwire; Boston Scientific Corp., Marlborough, Massachusetts, USA; Fig. 3 **c, d** ). The pancreatogram showed that the morphology of the APD was suitable for stent placement. A plastic stent (7 Fr, 8 cm) was then placed in the pancreatic duct ( Fig. 4 **a, b** ). Pancreatic juice was seen flowing out of the stent ( Video 1 ).

**Fig. 2:**
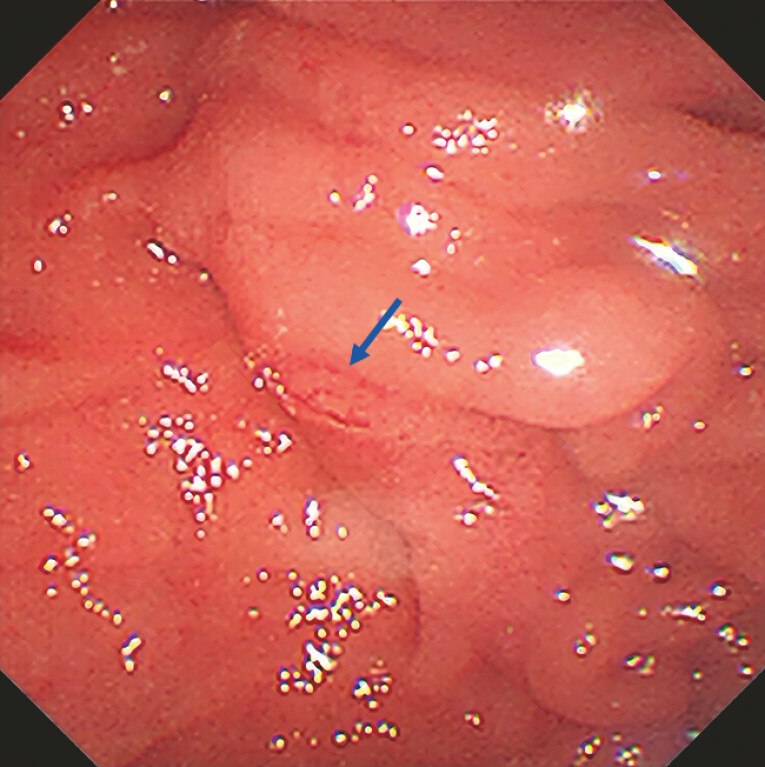
The minor papilla was not obvious under the microscope (the head of the blue arrow is shown as the main duodenal papilla).

**Fig. 3:**
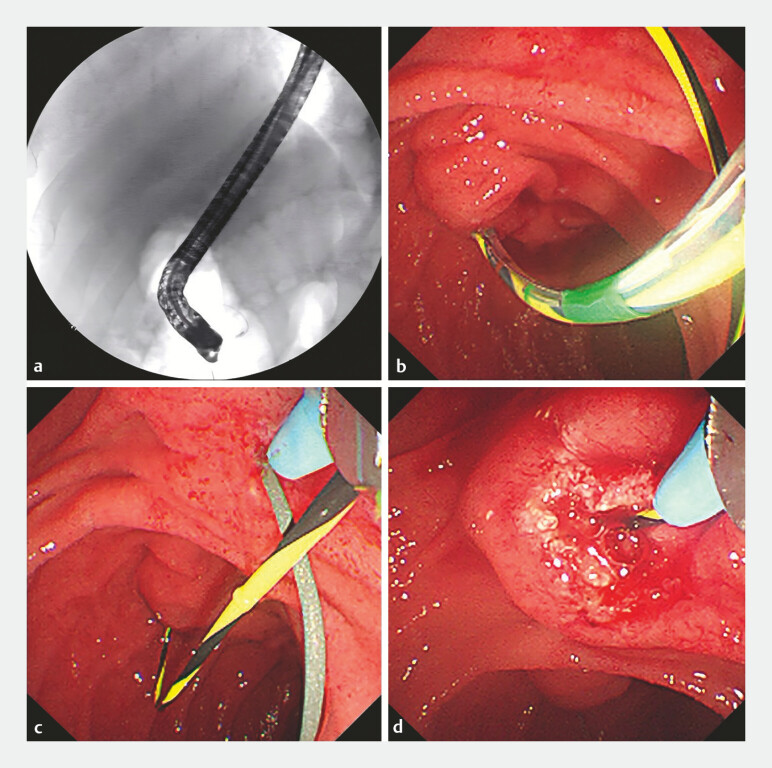
Guidewire and minor papilla sphincterotomy using the needle knife. **a, b** The guidewire in the MPD successfully passed through the minor papilla and coiled in the duodenal lumen. **c, d** Under the guidance of the guidewire, the approximate location of the minor papilla was found, and sphincterotomy was performed using the needle knife after pulling out the guidewire, and the guidewire intubated the minor papilla along the incision in the APD.

**Fig. 4:**
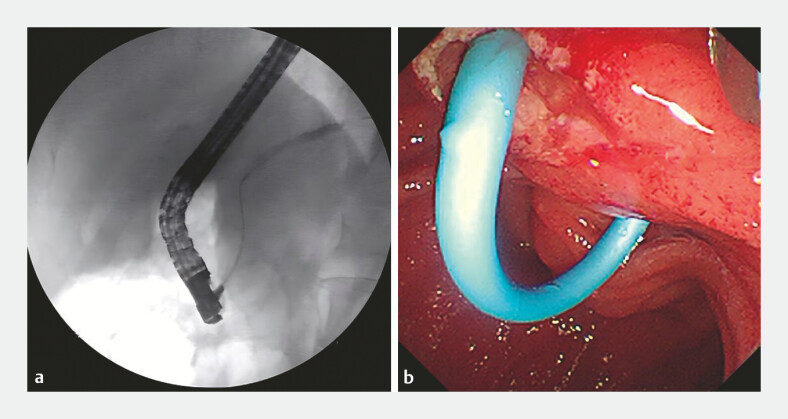
Pancreatogram showed that the morphology of the APD was suitable for stent placement. A plastic stent (8.5 Fr, 5 cm) was then placed in the pancreatic duct.

Under the guidance of a guide wire, the approximate location of the minor papilla was found.Video 1

Placing a pancreatic stent during endoscopic retrograde cholangiopancreatography or sphincterotomy of the minor papilla is the first-line treatment for pancreatitis with pancreatic divisum. This study proposed a new method, referred to as the reverse cannulation/needle-knife papillotomy of the minor papilla, of assisting the cannulation and sphincterotomy of the minor papilla in patients with pancreatitis and a slender branched pancreatic duct between the MPD and the APD, in whom direct cannulation of the minor papilla was difficult.

Endoscopy_UCTN_Code_TTT_1AR_2AC

## Notes

Endoscopy E-Videos https://eref.thieme.de/e-videos
*E-Videos* is an open access online section of the journal *Endoscopy* , reporting on interesting cases and new techniques in gastroenterological endoscopy. All papers include a high-quality video and are published with a Creative Commons CC-BY license. Endoscopy E-Videos qualify for HINARI discounts and waivers and eligibility is automatically checked during the submission process. We grant 100% waivers to articles whose corresponding authors are based in Group A countries and 50% waivers to those who are based in Group B countries as classified by Research4Life (see: https://www.research4life.org/access/eligibility/ ). This section has its own submission website at https://mc.manuscriptcentral.com/e-videos .
